# Do fixed orthodontic appliances cause halitosis? A systematic review

**DOI:** 10.1186/s12903-019-0761-1

**Published:** 2019-05-02

**Authors:** Salem Abdulraheem, Liselotte Paulsson, Sofia Petrén, Mikael Sonesson

**Affiliations:** 1 Department of Orthodontics, Faculty of Odontology, Malmö University, SE-20506 Malmö, Sweden; 2 Ministry of Health, Kuwait City, Kuwait

**Keywords:** Fixed orthodontic appliance, Halitosis, Systematic review

## Abstract

**Objective:**

To examine: (I) the current evidence of the impact of fixed orthodontic appliances on the development of halitosis in patients undergoing orthodontic treatment, and (II) the influence of different orthodontic bracket systems on halitosis.

**Material and methods:**

Three electronic databases (PubMed, Scopus, and Cochrane Library) were searched prior to March 15, 2018. The review was systematically conducted and reported according to the Cochrane Handbook and the PRISMA statement. Only Randomised Clinical Trials (RCTs) were considered. Selected full-text papers were independently assessed by four investigators and any disagreements were resolved by consensus. The Cochrane Handbook was used to grade the risk of bias and the quality of evidence was rated according to GRADE.

**Results:**

Out of 363 identified studies, three RCTs on halitosis and fixed orthodontic appliances met the inclusion criteria. The risk of bias in the three studies was rated as high and the quality of evidence was rated as very low.

**Conclusions/clinical implications:**

There is a lack of scientific evidence that subjects with fixed orthodontic appliances develop halitosis during treatment. Additional well-conducted RCTs with extended periods of assessment are needed as well as consensus concerning cut-off values for the diagnosis of halitosis.

## Background

Halitosis is defined as an unpleasant mouth breath arising from pathological, non-pathological, physiological or systemic conditions [1]. Halitosis is common, and up to 50% of the population is reported to be affected to various degrees [2]. Individuals with halitosis do not always notice the symptoms by themselves, which might result in an underestimation of its prevalence [3]. Several oral etiological factors for halitosis, such as tongue coating, specific microbes, poor oral hygiene, diseases such as gingivitis and periodontitis, along with smoking, have been identified [4]. Crucial to the development of halitosis is the generation of volatile sulphur compounds (VSCs) of certain bacteria, during their proteolytic degradation of amino acids in saliva, epithelium, gingival crevicular fluid, dental plaque, blood and food debris [5–10]. When a fixed orthodontic appliance is inserted, the area of plaque accumulation and the amount of generated proteins from gingival crevicular fluid and saliva will increase, which elevates the amount of available nutrients for the supra- and subgingival microorganisms, thus increasing the risk for halitosis [11]. It has also been discussed that the type of bracket system may influence the development of periodontal diseases during treatment. Pellegrini et al. [12] showed that self-ligated brackets (SLBs) had a lower negative impact on destructive biological events in the periodontium compared to conventional brackets (CBs) with elastomeric ligatures, which might have an impact on the development of halitosis. On the other hand, Pandis et al. [13] stressed that the opening and closing mechanisms of SLBs also accumulate plaque, which might increase the risk for adverse periodontal effects similar to CBs.

To detect halitosis, two fundamental approaches are available: the instrumental and the organoleptic methods [11, 14, 15]. The instrumental methods utilise electronic devices such as the Halimeter or Oral Chroma to measure the amount of volatile sulphur compounds (VSCs) [7, 9, 16, 17]. However, studies using the Halimeter used different cut-off values for the measurement of halitosis. Values below 100 ppb VSC or between 70 and 110 ppb VSC have been considered normal; the manufacturer of the Halimeter, on the other hand, states that values between 50 and 150 ppb VSC are normal [18, 19]. The organoleptic method is a subjective registration directly beside the mouth of the patient. The severity of the offensive odour of the patient’s breath is measured by using an organoleptic scoring system (OLS) [18]. Both approaches have been assessed and seem to correlate well in detecting halitosis [19].

The aims of the present systematic review were to examine: (I) the current evidence on the impact of fixed orthodontic appliances on the development of halitosis in patients undergoing orthodontic treatment, and (II) the influence of different orthodontic bracket systems on halitosis.

## Methods

### Protocol and registeration

This systematic review was made following the PRISMA-P Statement [20] and was registered in the National Institute of Health Research database with an appropriate protocol number (http://www.crd.york.ac.uk/PROSPERO) Protocol: CRD42017074854. The systematic review was systematically conducted and reported according to the Cochrane Handbook [21] and the PRISMA statement [22].

### Eligibility criteria

The eligibility criteria were based on PICOS: Population: healthy patients, without age restriction; Intervention: treatment with fixed orthodontic appliance; Control: no treatment with fixed appliance; Outcomes: development of halitosis registered by electronic devices (Oral Chroma, Halimeter) or Organoleptic scoring; Studies: Randomized Controlled Trials (RCTs). The exclusion criteria were studies on patients with cleft lip and palate and patients treated with removable appliances or clear aligners. Studies with a prospective control design, retrospective design, case reports, or experts’ opinions were also excluded.

### Information sources and search strategy

Three electronic databases (PubMed, Scopus, and Cochrane Library) were searched prior to March 15, 2018. One author (S.A) performed the search with assistance from the library staff at Malmo University. In addition, https://clinicaltrials.gov/ was checked for ongoing studies. The search terms were; ‘bad breath’, ‘oral odor’, ‘oral malodor’, ‘oral malodour’, ‘oral odour’, ‘Halitosis’, ‘Orthodontic Appliances’, ‘Orthodontic Appliance’, ‘Orthodontic Bracket’, ‘Orthodontic Brackets’, ‘Orthodontic Braces’, ‘fixed orthodontic appliances’ and ‘fixed orthodontic appliance’ in various meshwords and free-text combinations. The full search strategy is presented in Table 1. An additional search was performed using the keywords ‘adolescents and halitosis’ as a free text search in the PubMed database.

**Table 1:** Search strategy searching PubMed

Search block	
#1	Bad breath OR oral odor OR oral malodor OR oral malodour OR oral odour OR Halitosis OR “Halitosis” [Mesh] AND Orthodontic Appliances [Mesh] OR Orthodontic Appliances OR “Orthodontic Brackets”[Mesh] OR Orthodontic appliances OR “Orthodontic Brackets” OR Orthodontic Brackets OR Orthodontic Braces OR fixed orthodontic appliances OR fixed orthodontic appliance
#2	Adolescents AND Halitosis

**Fig. 1:**
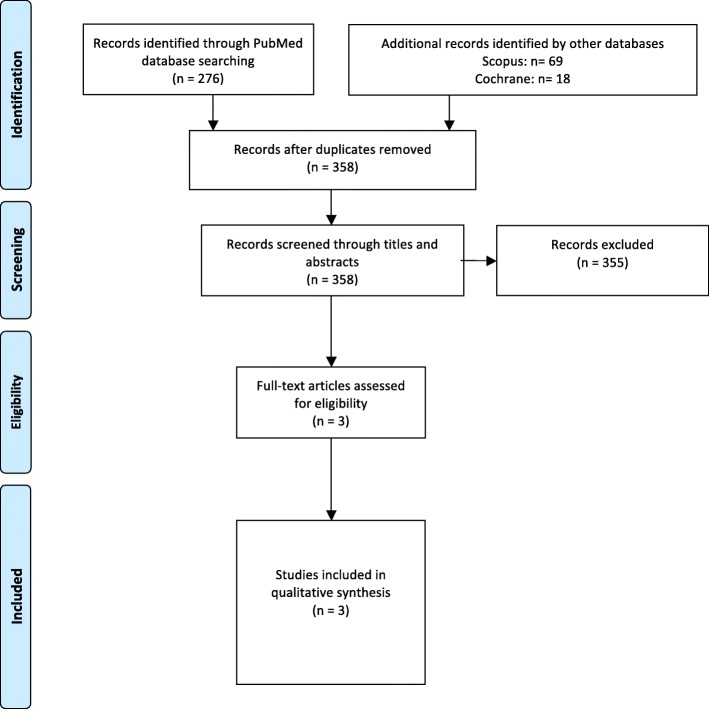
Flowchart of study selection performed according to the PRISMA guidelines

For each selected abstract, the information on related studies was checked in the database. Only papers published in English or Swedish were accepted. The reference lists of the accepted papers were searched manually for additional literature. A Prisma flowchart of the included and excluded studies is presented in Fig. 1.

### Study selection

Four reviewers (S.A, L.P, S.P, M.S) reviewed and analysed the abstracts of the eligible studies. The retrieved articles were chosen because they were full-text length, and were assessed by the four examiners independently. Any conflicts or disagreement between the reviewers were resolved by consensus.

### Data collection process and data item

Data collection from the full-text studies was performed by the reviewers (S.A, L.P, S.P, M.S) independently. A standardised pre-set protocol was used. The protocol included: (i) study characteristics (language, design, country, clinical setting); (ii) study population (age, sex, total sample, dropouts) and (iii) intervention and adverse effect (type of fixed appliance, extension of the fixed appliance, length of the follow-up, number of VSC or organoleptic registrations, observer experience, VSC measurement, other measurements that were related to halitosis, patient self-reporting of halitosis and outcome analysis).

### Risk of bias within and across the studies

The four reviewers individually assessed the quality of each study by using the Cochrane risk of bias assessment tool [21]. The following six domains were considered during the assessment: 1. random sequence generation, 2. allocation sequence concealment, 3. blinding of outcome assessment, 4. incomplete outcome data, 5. selective outcome reporting, and 6. other sources of bias. Each RCT was assigned an overall risk of bias; for example, low risk of bias if all key domains had low risk, high risk if more than one key domain had high risk, and unclear risk if more than one key domain had unclear risk.

The quality of evidence was rated using the GRADE tool [23]. Four categories were set: strong, moderate, low and very low.

## Results

### Study selection

The initial search identified 363 studies. After exclusion of irrelevant studies and removal of duplicates, three RCTs were left for full-text assessment (Fig. 1). All three RCTs were found in the PubMed database. The main reasons for exclusion were non-RCT design and irrelevant studies that did not deal with the topic of the present investigation. Additional information about the design of the investigations, mentioned as “unknown” in Table 2, was requested from the principal authors of the included studies. No answers were provided. The datasets used and/or analysed during the current study available from the corresponding author on reasonable request.

**Table 2:** Summarised data of the three studies included in the review

Study information First author Year Country	Study design Design Setting (n)	Sample (n) Initial Final Dropouts Reasons for dropouts	Interventions Groups, appliances, extension Number Gender (girls/boys) Age (mean yrs./SD) Age (range)	Halitosis examinations VSC measurement method Experience of the examiner(s) Time of measurement and mean value (SD/range)	Additional examinations	Outcomes
Kaygisiz 2015 [24] Turkey	- RCT - University, Orthodontic clinic	- 60 - Unknown - Unknown - Not applicable	Group I: SLB in both arches - 20, - Unknown, - 14.7 / 1.39 Group II: CB in both arches - 20, - Unknown, - 14.0 / 1.01 Group III: Control without appliance - 20 - Unknown −14.4 / 1.46	- Halimeter - One periodontist - Immediately before bonding: Group I: 76.85 ± 64.82 Group II: 53.20 ± 41.19 Group III: 34.87 ± 34.28 - One week after bonding: Group I: 62.15 ± 56.51 Group II: 64.9 ± 40.90 Group III: 43.93 ± 39.75 - Four weeks after bonding: Group I: 48.80 ± 38.94 Group II: 49.15 ± 36.19 Group III: 42.93 ± 38.00 - Eight weeks after bonding: Group I: 49.20 ± 21.38 Group II: 43.10 ± 31.05 Group III: 47.40 ± 25.17	Plaque Index Gingival index Pocket depth Bleeding on probing Tongue coating index	No statistically significant difference between pre-bonding and post-bonding VSC values No statistically significant difference between the two groups receiving fixed appliances and the control group The SLB was not advantageous over the conventional brackets None of the groups’ VSC mean value reached halitosis cut offs
Nalcaci 2014 [25] Turkey	- RCT - University	- 46 - Unknown - Unknown - Not applicable	Group I: SLB Unknown number of arches - 23 - 11/12 - 14.48/ 1.27 Group II: CB unknown number of arches - 23 - 13/10 - 13.38/ 1.61	- Halimeter - Periodontist - Before placement of brackets: Group I: 43.70 ± 2.20 Group II: 41.78 ± 1.77 - One week after bonding: Group I: 56.00 ± 2.96 Group II: 60.32 ± 2.94 - Five weeks after bonding: Group I: 59.10 ± 3.62 Group II: 88.2 ± 3.85	Plaque index Gingival index Bleeding on probing Bacterial counts	Statistically significant increase of VSC mean values between pre-bonding and post-bonding measured after 1 and 5 weeks of bonding. Five weeks after bonding, SLB had significantly lower VSC mean values than CB when compared. None of the groups’ VSC mean value reached halitosis cut offs
Babacan 2011 [26] Turkey	- RCT - University orthodontic department	- 44 - 41 - 3 - Missing measurements	Group I: CB in both arches - 21 - 12/9 - 13.05/ 1.48 Group II: Control without appliance - 20 - 11/9 - 13.70/ 1.61	- Halimeter - Periodontist - Before placement of brackets: Group I: 58.55 ± 13.77 Group II: 58.15 ± 14.32 One week after bonding: Group I: 81.00 ± 17.15 Group II: 60.85 ± 12.05 - Four weeks after bonding: Group I: 94.70 ± 12.31 Group II: 61.35 ± 15.41	Plaque index Gingival index	Statistically significant increase of VSC mean values between pre-bonding and post-bonding measured after 1 and 4 weeks of bonding. Statistically significant difference between the two groups receiving fixed appliances and the control group None of the groups’ VSC mean value reached halitosis cut offs

### Study characteristics

The characteristics of the three included studies are presented in Table 2. The studies were RCTs published between 2011 and 2015 [24–26] and were conducted in Turkey. All the studies assessed halitosis using a Halimeter device, measuring the amount of VSC in the oral cavity before the bonding of the fixed orthodontic appliance and during check-ups, which ranged between 1 and 8 weeks after bonding. Two studies [24, 26] included a control group without orthodontic fixed appliances, and one study [25] compared two types of orthodontic appliances: conventional fixed orthodontic appliances (CB) and self-ligating fixed orthodontic appliances (SLB).

### Risk of bias within and across the studies

**Fig. 2:**
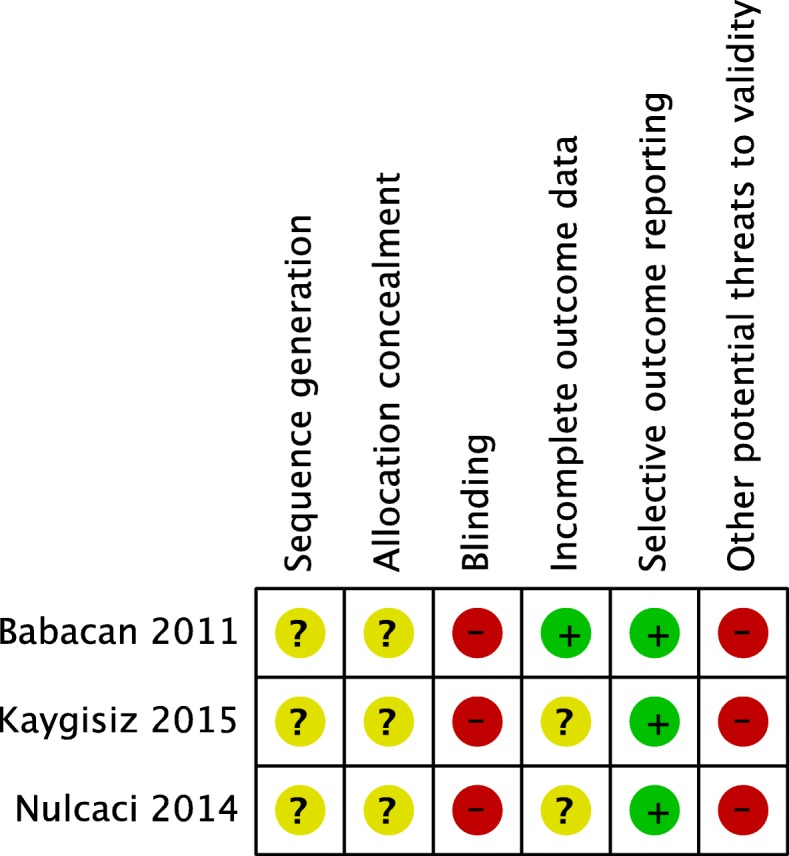
The four assessors’ judgements using the Cochrane risk of bias assessment tool [21]. The domains were assessed as low, high or unclear risk of bias (marked as green, yellow and red). All studies were assigned as an overall risk of bias

All the included studies were scored as having an unclear risk of bias in the sequence generation and allocation concealment domains. Regarding the blinding domain, the studies did not perform blinding for any of the blinding categories (patients, trial staff and/or outcome assessors); thus all trials were considered to have a high risk of bias in this domain. All the included studies were scored as high risk of bias in relation to potential threats to the validity domain, due to design specific risks of bias as only first period of treatment data was available (Fig. 2). The quality of evidence across the studies was assessed as very low for all the aims in this systematic review (⊕ΟΟΟ).

### The effect of orthodontic fixed appliance on halitosis

The results of the studies were contradictory. Two of the included studies [25, 26] showed a statistically significant increase of VSC levels 1 week after insertion of fixed orthodontic appliance, compared to before insertion. The VSC levels were also statistically significantly higher during the check-up 4 weeks [26] and 5 weeks [25] after insertion of the appliance. In contrast, Kaygisi et al. [24] showed no statistically significant difference in VSC mean values before insertion and after 1, 4 and 8 weeks of treatment [24].

### The effect of orthodontic fixed appliance on halitosis compared to controls

Two of the three included studies had a control group without a fixed orthodontic appliance, but the results were contradictory. One of the studies [26] showed statistically significantly higher VSC levels in the subjects with fixed appliance 1 and 4 weeks after insertion of the appliance (mean 81.00, SD 17.15 mean 94.70, SD 12.31, respectively) compared to the control subjects (mean 60.85, SD 12.05 and mean 61.35, SD 15.41, respectively). The study by Kaygisiz et al. [24] compared three groups: 1) CB, 2) SLB, and 3) a control group, and did not found any statistically significant differences in VSC levels between the groups after 1, 4 and 8 weeks of follow-ups.

### The effect of different bracket systems on halitosis

Two of the three included studies compared the development of halitosis in patients with self-ligating brackets (SLB) or conventional brackets (CB), and the results were contradictory [24, 25]. The study of Kaygisiz et al. [25] showed no statistically significant difference between the CB and the SLB groups concerning VSC level before bonding, and 1, 4 and 8 weeks after bonding [24]. The study by Nalcaci et al. 2014 showed that, after 5 weeks of treatment, the SLB group had a statistically significantly lower VSC level (mean 59.10, SD 3.62) compared to the CB group (mean 88.2, SD 3.85). In addition, no statistically significant difference in VSC levels between the groups was shown before and 1 week after insertion of the appliance [25].

The risk of bias in the three studies was rated as high (Fig. 2). Consequently, the quality of evidence was rated as very low for the three aims’ questions (⊕ΟΟΟ).

## Discussion

This systematic review shows that there is a lack of evidence regarding the development of halitosis during treatment with fixed orthodontic appliances. To the best of our knowledge, this is the first systematic review of the quality of available evidence on the development of halitosis in patients with fixed orthodontic appliances. Halitosis in the general population has been thoroughly investigated; but the development of halitosis in patients with fixed orthodontic appliances seems to be poorly investigated. All the included RCTs were evaluated as having a high risk of bias, following assessment using the Cochrane risk of bias assessment tool [21]. Sequence generation and allocation concealment were assessed as being unclear in all the included studies. The included studies did not perform any blinding, and are thus assessed as having a high risk of bias. Other potential threats to the validity domain were also found to have high risk of bias due to the short follow-up period. Sufficient follow-up periods are required as the biofilm responsible for the development of halitosis often has a mature composition of microbiota, due to changes in available nutrients, which takes more than 3 months to develop [27]. This indicates that all the included RCTs underestimated the VSC amount measured by Halimeter since all the studies only followed the patient for a period of 1–8 weeks and none reached at least 3 months of follow-up. It is also important to note that all patients included in the trials were subjected to the Hawthorne effect. The Hawthorne effect is defined as changes in patients’ or therapists’ behaviour when involved in a trial because of increased knowledge or interest or due to being aware of observation [28]. A recent systematic review [29] was assessed to elucidate whether the Hawthorne effect exists, to explore the conditions it may exist under, and to estimate the size of any such effect. The study confirmed the presence of such a phenomenon and that it causes overoptimistic results (false positive bias) in RCTs. The review also concluded that the Hawthorne effect is almost non-existent once participants have had more than 6 months’ involvement in the trial [29]. In this systematic review, the participants were involved in the trial for a maximum of 8 weeks, which means that the Hawthorne effect may have influenced the results.

An additional factor that might influence the VSCs’ mean value and the results of this sytematic review is patients’ adherence to food instructions. Different types of food can influence the development of halitosis, especially spicy foods and onions. One strategy to reduce this factor could be to perform a detailed food registration and document the patients food intake. All three of the included studies used the Halimeter method to measure halitosis and presented the values of the VSC. None of the included studies used the organoleptic method. The validity of this later method has been assessed with positive results and should preferably be used in studies on halitosis.

Three RCTs were included to evaluate the effect of fixed orthodontic appliances on VSC mean values in the oral cavity and whether the appliances cause halitosis. Pre- and post-bonding measurements were compared. Two studies showed that the insertion of fixed orthodontic appliances caused an increase in VSC mean values but without causing halitosis, as the mean values were below the cut-off value [25, 26]. However, one RCT showed the opposite results [24]. Based on the included RCTs, fixed orthodontic appliances cause an increase of VSC mean values in the oral cavity, but without reaching the halitosis cut-off values.

Two studies evaluated the effect of different fixed orthodontic appliance on VSC mean values and whether the appliances caused halitosis when the patients were compared to controls. One RCT presented statistically significantly higher VSC values for the subjects with fixed orthodontic appliances compared to the controls, but the presented mean VSC values were below the halitosis cut-off values [25]. On the other hand, one RCT showed the opposite [24].

Another important finding is that different types of fixed orthodontic appliances seem to accumulate similar amounts and types of plaque. Thus, no evidence regarding which appliance reduces the risk of halitosis seems to exist. The present systematic review was unable to provide scientific support for the relationship between treatment with conventional or self-ligated fixed orthodontic appliances and the development of halitosis.

Different cut-off values for halitosis have been described in previous studies on the Halimeter and from the manufacturer [18, 19, 27, 30]. This could indicate that, even if there were statistically significant differences in the VSC values between the subjects with fixed appliance and controls, all subjects were within the normal VSC range, and thus did not have halitosis. This means that, according to the available evidence, subjects with fixed orthodontic appliances may have an increase in VSC levels during the fist one and a half months of treatment, but not fully developed halitosis.

The strength of this systemtic review is that four individual reviewers searched and analysed the studies. Furthermore, the search was performed in three main databases as well as the reference lists of the included studies, which minimises the possibility of missing any studies. Finally, including only RCTs strengthened this systematic review since doing so provided us with the highest level of evidence. A possible limitation might be that only studies written in English or Swedish were included. Another limitation is that all the results of the present systematic review are based on a limited number of RCTs, performed in one country (Turkey), and all were rated as having a high risk of bias. The Halimeter has some limitations, as mentioned above. Therefore, well-designed studies on the organoleptic method to assess halitosis throughout the orthodontic treatment period are warrented. Furthermore, it is recommended that future studies conduct a proper power calculation and use Intention To Treat (ITT) analysis when assessing the results.

## Conclusion

1 - Based on the very low quality of evidence:

A - Fixed orthodontic appliances cause an increase in VSC mean values but without reaching the halitosis cut-off values when pre- and post-bonding values are compared.

B - Fixed orthodontic appliances do not increase VSC mean values or cause halitosis, when patients with fixed orthodontic appliances are compared to the control group.

C - There is no difference between conventional brackets and self-ligating brackets in VSC mean value increase or the development of halitosis.

2 - Based on the available literature, there is a lack of reliable evidence showing that subjects with conventional or self-ligated fixed orthodontic appliances develop halitosis during treatment. Further well-conducted controlled clinical trials with extended follow-ups and consensus concerning cut-off values for the diagnosis of halitosis are needed to establish best scientific evidence.

## Abbreviations

CBs: Conventional brackets
OLS: Organoleptic scoring system
RCTs: Randomised controlled trials
SLB: Self-ligated brackets
VSC: Volatile sulphur compounds
